# Charge‐State‐Dependent Energization of Suprathermal Ions During Substorm Injections Observed by MMS in the Magnetotail

**DOI:** 10.1029/2020JA028144

**Published:** 2020-09-29

**Authors:** S. T. Bingham, I. J. Cohen, B. H. Mauk, D. L. Turner, D. G. Mitchell, S. K. Vines, S. A. Fuselier, R. B. Torbert, J. L. Burch

**Affiliations:** ^1^ The Johns Hopkins University Applied Physics Laboratory Laurel MD USA; ^2^ Southwest Research Institute San Antonio TX USA; ^3^ Department of Physics and Astronomy University of Texas at San Antonio San Antonio TX USA; ^4^ Space Science Center University of New Hampshire Durham NH USA

**Keywords:** injections, magnetotail, MMS, plasma sheet, ion energization, substorms

## Abstract

Understanding the energization processes and constituent composition of the plasma and energetic particles injected into the near‐Earth region from the tail is an important component of understanding magnetospheric dynamics. In this study, we present multiple case studies of the high‐energy (≳40 keV) suprathermal ion populations during energetic particle enhancement events observed by the Energetic Ion Spectrometer (EIS) on NASA's Magnetospheric Multiscale (MMS) mission in the magnetotail. We present results from correlation analysis of the flux response between different energy channels of different ion species (hydrogen, helium, and oxygen) for multiple cases. We demonstrate that this technique can be used to infer the dominant charge state of the heavy ions, despite the fact that charge is not directly measured by EIS. Using this technique, we find that the energization and dispersion of suprathermal ions during energetic particle enhancements concurrent with (or near) fast plasma flows are ordered by energy per charge state (*E*/*q*) throughout the magnetotail regions examined (~7 to 25 Earth radii). The ions with the highest energies (≳300 keV) are helium and oxygen of solar wind origin, which obtain their greater energization due to their higher charge states. Additionally, the case studies show that during these injections the flux ratio of enhancement is also well ordered by *E*/*q*. These results expand on previous results which showed that high‐energy total ion measurements in the magnetosphere are dominated by high‐charge‐state heavy ions and that protons are often not the dominant species above ~300 keV.

## Key Points

- In the magnetotail during injections, the charge states of suprathermal He and O ions can be inferred with a correlation analysis
- Energization of ionospheric and solar wind ions during injections in the magnetotail is remarkably coherent and ordered by charge state
- The highest energy ions (≳300 keV) observed are heavies of solar wind origin and reach higher energies due to their higher charge states

## 1 Introduction

Energetic ions from a few keV to hundreds of keV comprise the terrestrial ring current and nightside plasma sheet, which play important roles in contributing to the energy density of the magnetosphere (e.g., Jordanova et al., 2020). Understanding the transport and energization of the thermal and suprathermal ion populations has been a longstanding question in magnetospheric physics. The complexity in tracking and understanding ion dynamics in the magnetosphere comes from the ions' multisource nature, multispecies composition, and competing energization and loss processes. The most prevalent ion species in the plasma sheet and ring current are protons, oxygen, and helium (e.g., Young et al., 1982). Often, in the absence of observations of composition, ions in the ring current and plasma sheet are assumed to be protons, due to their typical dominance compared to the other species in many regions of the magnetosphere. Both the ionosphere (Ghielmetti et al., 1978; Shelley et al., 1972) and solar wind (Axford, 1970; Balsiger et al., 1980; Rauch & Roux, 1982) are sources for ions in the magnetosphere. Consequently, heavy ions of different charge states are observed throughout the magnetosphere (e.g., Allen et al., 2016, 2017; Gloeckler & Hamilton, 1987). However, when only composition measurements that do not distinguish charge state are available, it is common to assume that heavy ions are singly charged. This assumption, while often valid for low energies, can lead to vastly different interpretations of ion heating and energization of the suprathermal populations within the magnetosphere.

Due to the range of different temporal or spatial scales in which different forms of energization can occur, the range of *mass/charge (m*/*q*) values found in the magnetosphere has long been used as a tracer to study the origin and acceleration of magnetospheric plasma (e.g., Axford, 1970; Balsiger et al., 1980; Blake & Fennell, 1981; Cornwall, 1972). Previous observations have shown that the oxygen energy density can be comparable to or exceed the proton energy density in the ring current and plasma sheet during geomagnetically active times (Gkioulidou et al., 2016; Kunihiro Keika et al., 2013; Maggiolo & Kistler, 2014; Mouikis et al., 2019; Young et al., 1982; Zhao et al., 2015). Moreover, the profiles of energy density versus radial distance from the Earth are not the same for oxygen and protons (e.g., Lui, 2003; Ohtani et al., 2011), which naturally leads to questions about species‐dependent energization processes. Kronberg et al. (2015) reported using observations from Cluster/RAPID (Wilken et al., 1997) that plasma sheet ion intensities at high energies (>274 keV) were mass dependent, with oxygen intensity enhancements being significantly greater than protons. Additionally, Luo et al. (2014) showed with RAPID data that oxygen ions had a harder power law spectrum than protons between 150 keV and 1 MeV at radial distances between 16 and 20 *R*_*E*_. It should be noted that RAPID is unable to distinguish ion charge state. Cohen et al. (2017) similarly found with the Energetic Ion Spectrometer (EIS) instrument on the Magnetospheric Multiscale (MMS) mission that for *L* shells between 7.5 and 16.5, oxygen had a harder spectrum than protons and that oxygen intensities dominated proton intensities at energies above ~150–220 keV during both quiet and geomagnetically active times. Like RAPID, EIS does not directly measure charge state. Cohen et al. (2017) contended that if one assumed the high energy oxygen was actually O^6+^ instead of O^+^, the power law spectrum in intensity would be well ordered by *E/q*. This power law spectrum would align with previous high energy plasma sheet observations of solar wind ions reported by Gloeckler and Hamilton (1987) using the CHEM and SULEICA instruments, which could differentiate charge state, on AMPTE/CCE (see Dassoulas et al., 1985; Gloeckler et al., 1985) and on AMPTE/IRM (see Häusler et al., 1985; Möbius et al., 1985). Additionally, observations from Polar comparing O^+^ and O^6+^ flux versus energy found that O^6+^ had comparable flux values and a harder spectrum than O^+^ at the highest energies (~200 keV; Allen et al., 2017).

The bounce‐averaged guiding center description of particle motion can be expressed as

$$\frac{dx}{\mathrm{dt}}=v_{E}+\frac{{\mathrm{KE}}_{⊥}}{q}v_{\mathrm{gc}},$$

where ***v***_*E*_ is the *E* × *B* drift and $\frac{{\mathrm{KE}}_{⊥}}{q}v_{\mathrm{gc}}$ is the energy per charge‐state‐dependent gradient curvature drift. This equation is often used to explain the energy per charge state ordering of observed spectra (e.g., Kistler et al., 1989; and references therein). Thus, if there are no changes to the fields on temporal or spatial scales smaller than the respective ion scales, then O^+^, H^+^, He^++^, and O^6+^ with the same initial *KE*_⊥_/*q* have the same drift trajectories and a *q*‐dependent energization. For example, a simple analytic Volland‐Stern description of the magnetosphere with a constant cross‐tail electric field and dipolar magnetic field will give drift paths, energization, and Alfvén layers which are a function of a particle's initial *μ/q*, where *μ* is the first adiabatic invariant (Stern, 1975; Volland, 1973). However, ion injections or rapid enhancements of energetic ions, which are important for populating the ring current and plasma sheet, are thought to be generated by processes that potentially violate adiabaticity (e.g., K. Keika et al., 2018). An example of such a process is magnetic reconnection in the magnetotail, which generates flow bursts (bursty bulk flows—BBF) and associated electric fields concurrent with the field dipolarizations (e.g., Birn et al., 2012; Gabrielse et al., 2012, 2014). Observations of injections often feature ions energized to hundreds of keV and even up to a few MeV. Since potential drops of that magnitude are not anticipated in the magnetosphere, the adiabatic nature and causal mechanisms of ion energization have been the subject of ongoing debate.

Mitchell et al. (2018) showed that a simple injection model of an azimuthally confined radial flow with an associated azimuthal electric field can adiabatically energize singly charged ions up to ~200–300 keV, He^++^ up to 400–600 keV, and O^6+^ above an MeV. They found that these modeling results well matched the high energy composition observations from the Radiation Belt Storm Probes Ion Composition (RBSPICE) instrument on board the Van Allen Probes at radial distances <5.8 *R*_*E*_ (like RAPID and EIS, RBSPICE does not distinguish charge state; Mitchell et al., 2013). Mobius et al. (1987) and Kistler et al. (1990) reported AMPTE/CCE/CHEM and AMPTE/IRM/SULEICA observations of the presubstorm and postsubstorm plasma sheet where the suprathermal ion enhancements featured an *E/q* hardening of the spectrum. Their results were replicated by a test‐particle‐in‐global‐magnetohydrodynamic (MHD) model (Sachsenweger et al., 1989). Ukhorskiy et al. (2018) studied ion energization in a three‐dimensional global MHD test particle simulation of ion trapping within magnetic islands formed by sharp magnetic field structure in dipolarizations fronts (DFs) traveling earthward from ~17 to ~6 *R*_*E*_ in the tail. They showed that ion acceleration and trajectories were well ordered by *E/q*, despite the violation of the first adiabatic invariant during the energization. However, Delcourt and Sauvaud (1994) and Delcourt et al. (1997) simulated test particles in a dipolarizing magnetic field and found that certain initial conditions could lead to a nonadiabatic energization which was mass dependent and thus not ordered by *E*/*q*. In a statistical study of energetic ion enhancements during dipolarizations from GEOTAIL/EPIC/STICS (which can resolve charge state; Williams et al., 1994), Ono et al. (2009) found separate cases in which either H^+^ or O^+^ had a harder suprathermal (~10–240 keV) spectrum, or greater energization; this result was hypothesized as being a consequence of turbulent wave frequencies observed to span both H^+^ and O^+^ in situ gyrofrequencies within BBFs. In a test particle simulation of ions interacting with stochastic electromagnetic perturbations within the current sheet, Catapano et al. (2017) found that suprathermal ion energization was strongly dependent on and proportional to charge state and only weakly dependent on ion mass (∝*m*^1/5^).

Despite extensive studies of ion energization there is still ambiguity on the roles of different acceleration mechanisms. A contributing factor to this ambiguity is a lack of understanding of the composition and charge state of suprathermal ions, particularly during active energization events like injections and BBFs. In this work we emphasize the importance of understanding the heavy ion charge state in the magnetosphere. Using a method first introduced by Mitchell et al. (2018) for the innermost regions using Van Allen Probes, we demonstrate that the dominant heavy ion charge state can be obtained from the MMS mission over broad regions of the magnetotail during energetic ion injection events using only observations of mass composition. In the present study we examine in some detail three specific events of dynamic energization of energetic ions in the magnetotail. Two cases are presented from ~7–10 *R*_*E*_ and one case is presented from ~25 *R*_*E*_. These three cases are qualitatively representative of the more than 40 cases that we have examined.

## 2 Instrumentation and Methodology

The MMS mission consists of four identically instrumented spacecraft (1–4) which were launched on 13 March 2015 (Burch et al., 2016). The four spacecraft maintain a tight tetrahedral spacing of 10–100 km over elliptical, low‐inclination orbits. The cluster of observatories has a perigee of ~2,500 km altitude and a geocentric apogee which has ranged from ~12–25 *R*_*E*_, depending on the mission phase. The apse line precesses ~360°/year. This orbit provides opportunities to observe particle injections across a range of radial distances from the Earth. The EIS is part of the Energetic Particle Detector investigation (EPD; Mauk et al., 2016) on board each MMS spacecraft. EIS measures energetic ion distributions in energy, pitch angle, and mass composition for protons (~20 keV to 1 MeV), helium (~60 keV to 1 MeV), and oxygen (~130 keV to 1 MeV). During particular regions of interest, MMS obtains higher resolution “burst” data, which has a Δ*E*/*E* of ~10%, compared to ~35% during typical “survey” data acquisition periods. It should be noted that EIS does not distinguish between carbon, nitrogen, and oxygen (CNO). However, since oxygen is typically the most abundant of this group, the CNO group is henceforth referred to as oxygen (e.g., Christon et al., 1994). As noted earlier, since EIS only comprises a time‐of‐flight (TOF) and solid‐state detector (SSD) system, it does not distinguish charge state. For example, incident 300 keV O^+^ and 300 keV O^6+^ both have the same TOF and input SSD energy, and thus both register as counts of 300 keV oxygen. Such “TOF‐SSD” systems have been used to measure energetic particle intensities on many other recent missions (e.g., Clark et al., 2016), and it is sometimes forgotten that heavy ions with multiple charge states will be measured by these instruments. Previous studies have shown that, because of the *E/q* dependence of the magnetic gradient and curvature drifts (Schulz & Lanzerotti, 1974), the timing of injections and drift echoes are typically temporally ordered by energy/charge state (where fluxes increase at the highest *E/q* values first, followed by those at lower *E/q* values). This temporal ordering can be used to deduce the dominant charge state for a heavy ion energy channel when compared to a proton energy channel (Blake et al., 1983; Blake & Fennell, 1981; Sibeck et al., 1988). Using the RBSPICE instrument (Mitchell et al., 2013) on board the Van Allen Probes spacecraft, Mitchell et al. (2018) used a similar technique to deduce the most likely heavy ion charge state in the inner magnetosphere (<5.8 *R*_*E*_) when only ion mass was known. RBSPICE is nearly identical in design to EIS and provides similar measurements. Mitchell et al. (2018) used the correlation between observed count rates in proton energy channels with helium and oxygen energy channels to infer the dominant heavy ion charge state for a given energy bin during periods where RBSPICE observed large energy‐dispersed ion injections over long periods of time (e.g., their Figures 7 and 8). In the present study, we employ a similar method to infer the dominant heavy ion charge state for a given heavy ion energy channel during substorm‐related energetic particle enhancements observed by EIS over vast regions of the magnetotail inaccessible to RBSPICE. Examples and greater details of this method will follow in the subsequent section.

The vector magnetic field on MMS is measured by the onboard fluxgate magnetometer (FGM) instrument (Russell et al., 2016). The Hot Plasma Composition Analyzer (HPCA; Young et al., 2016) and the Fast Plasma Investigation (FPI; Pollock et al., 2016) instruments are used to produce the ion moments, providing the ability to estimate the ion flow speeds. All moments calculated by FPI and HPCA are provided as a Level 2 data products (see Acknowledgements for data sources). HPCA resolves mass composition and *E*/*q* for energies ≲40 keV, while FPI cannot distinguish ion species and measures the total ion population ≲30 keV. During the events presented here, solar wind and geomagnetic index data comes from the NASA OMNI data set (King & Papitashvili, 2005).

## 3 Observations

**Figure 1:**
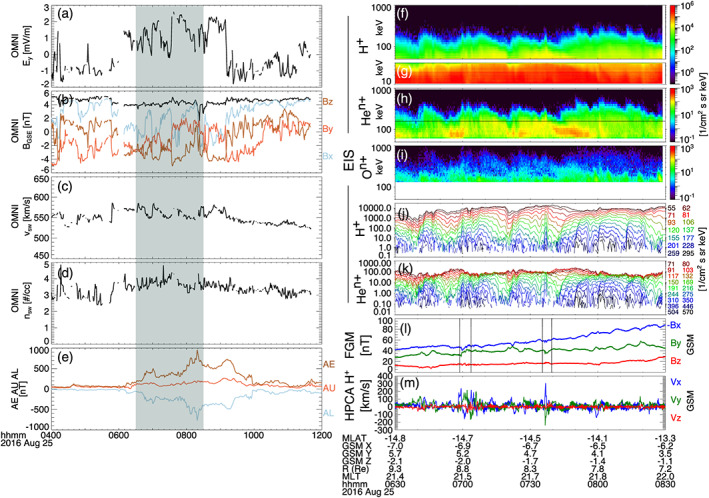
Solar wind observations from the OMNI database in GSE coordinates (a–d), auroral electrojet *AE*, *AU*, and *AL* indices (e), and MMS observations (f–m) of energetic ion enhancements during substorm activity. Solar wind (a) *y* component of electric field, (b) vector magnetic field, (c) speed, and (d) density. Energy spectra of differential flux for (f) EIS (TOF) H^+^, (g) EIS (xPH) H^+^, (h) EIS He^n+^, and (i) EIS O^n+^. Line spectra of differential flux for (j) EIS (TOF) H^+^ and (k) EIS He^n+^. (l) Vector magnetic field from FGM in GSM coordinates (note that since *Bx* < 0, −*Bx* is plotted for better visual comparison) and (m) H^+^ flow speed from HPCA in GSM coordinates. Panels (f)–(k) are combined spectra from MMS 2–4. Panels (l) and (m) are from MMS 3. Vertical black lines in (l) mark dipolarization‐like signatures.

We begin by showing observations of energetic particle enhancements by MMS during substorm activity on 25 August 2016. Figure 1 shows the solar wind conditions from the OMNI data (Figures 1a–1d), substorm auroral indices (*AE*, *AU*, *AL*; Figure 1f) from 04:00–12:00 UT, and MMS observations (Figures 1g–1m) from 06:30–08:30 UT. At ~06:00 UT, as the IMF *B*_*y*_ and *B*_*z*_ turn slightly negative and the solar wind electric field (−*v*_*x*_ × *B*_*z*_) is enhanced the geomagnetic auroral indices are also enhanced. From ~06:00–08:00 UT, *AE* transitions from ~70 to ~970 nT, indicating a significant substorm. During this time the MMS constellation was on the inbound portion of its orbit, moving from ~9.7 to 7.8 *R*_*E*_ at a magnetic local time (MLT) of 21.5 hr. Figures 1f–1i show energy spectrograms of the spin‐averaged (~20 s) omnidirectional differential flux (intensity) observed by EIS for 40–1,000 keV protons (ExTOF product), 10–40 keV protons (PHxTOF product), 50–1,000 keV helium, and 130–1,000 keV oxygen ions, respectively. Since the spatial separations of the MMS spacecraft are small (≲50 km) compared to the gyroradii of the ions (e.g., ~600–1,000 km for 100 keV protons in the observed magnetic field), and EIS observations from each available spacecraft show essentially identical features, the intensities shown are a combination of EIS data from MMS 2–4 (no ion products are available from EIS on MMS 1 during this event). Figures 1l and 1m show the local magnetic field observed by the FGM and the proton flow speeds observed by HPCA, respectively, from MMS 3 in GSM coordinates. Since *B*_*x*_ < 0, −*B*_*x*_ is plotted for a better visual comparison of changes to the component magnitudes. Concurrent with enhancements to the high energy particle fluxes, fast flows between −200 and +300 km/s are observed by HPCA. As indicated by changes to the magnetic field, dipolarization‐like signatures are observed at 07:00 and 07:36 UT (marked with vertical lines in Figure 1l). To better depict the energy dispersion observed in the ion enhancements, Figures 1g and 1h are line plots of the differential flux for each EIS proton and helium energy channel, respectively. The energies for these channels are color coded from low energy (dark blue) to high energy (red), where the corresponding energy of the channel in keV is to the right of the panel.

One can see that, typically, higher energies are enhanced slightly earlier in time before lower energies, and that enhancements for high energies are sustained for shorter periods of time than for lower energies. Additionally, flux enhancements for helium and oxygen register at higher energies than those observed for protons. For example, the flux enhancements of protons extend to ~350 keV, compared to ~700 keV for helium and up to the highest energy channel, ~1 MeV, for oxygen. If one were to observe this series of energetic ion enhancements with an instrument which did not differentiate ion species, it might seem reasonable to assume that protons were the dominant species in each energy channel. This would require extreme electric potentials for H^+^ to be energized up to these very high energies. With the analysis provided here, we know such large electric potentials are not required. As described earlier, EIS does not distinguish the charge state of the heavy ions, only the mass composition. If one were to assume that the helium and oxygen ions observed are singly charged, then it would require similar extreme potentials to reach these highest energies. Assuming that helium and oxygen are singly charged would also require an explanation for the extreme mass dependence of the energization. Instead, we will follow the example of Mitchell et al. (2018) to show that we can leverage the *E*/*q* dependence of the flux response during an injection to deduce the most likely dominant charge state for a given energy channel. This analysis shows that the preferential energization of these very high energy (>350 keV) heavy ions is primarily due to their higher charge state.

**Figure 2:**
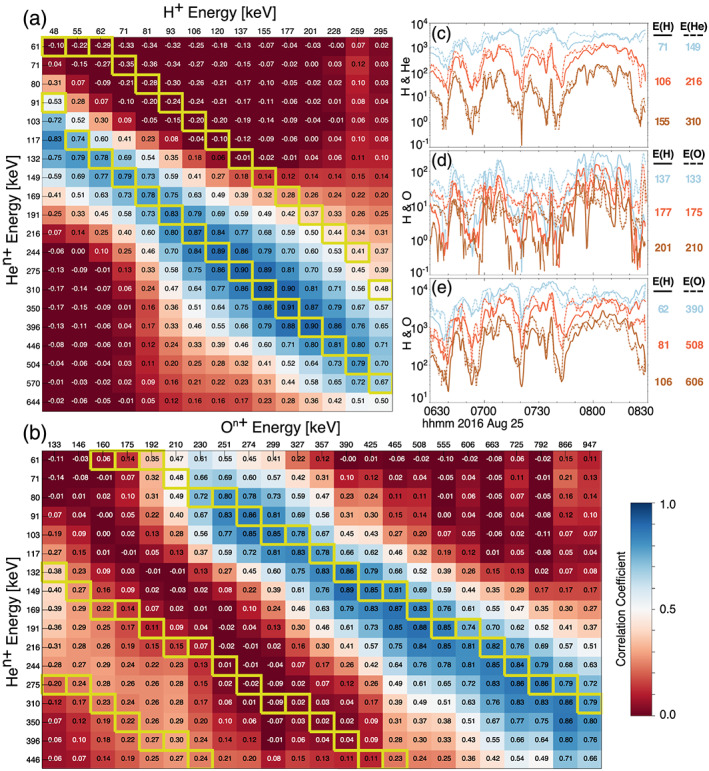
Panel (a) depicts correlation coefficients of time histories of EIS H and He flux between different energy channels of each species during the time period shown in Figure 1. Boxes with yellow outlines highlight energies where *E*_He_ = 2 * *E*_H_ and *E*_He_ = *E*_H_, which would indicate helium being He^++^ or He^+^, respectively. Similarly, Panel (b) depicts correlation coefficients between He and O, and yellow boxes highlight *E*_He_ = 2 * *E*_O_, *E*_He_ = *E*_O_, and 3 * *E*_He_ = *E*_O_, which with the inference from Panel (a) that helium is double ionized (He^++^) would signify oxygen charge states of 1, 2, or 6, respectively. Panel (c) shows the flux time histories of proton (solid lines) and helium (dashed lines) ions for energies *E*_He_ = 2 * *E*_H_. Each helium flux has been multiplied by a constant factor to visually compare its correlation with the proton fluxes. Similarly, Panel (d) shows proton (solid lines) and oxygen (dashed lines) fluxes for *E*_H_ = *E*_O_, and Panel (e) shows fluxes for 6 * *E*_H_ = *E*_O_.

**Figure 3:**
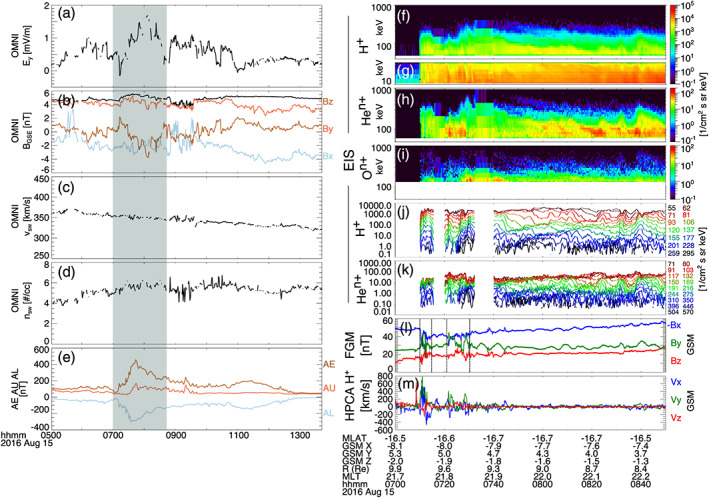
Solar wind observations from the OMNI database in GSE coordinates (a–d), auroral electrojet *AE*, *AU*, and *AL* indices (e), and MMS observations (f–m) of energetic ion enhancements during substorm activity. Solar wind (a) *y* component of electric field, (b) vector magnetic field, (c) speed, and (d) density. Energy spectra of differential flux for (f) EIS (TOF) H^+^, (g) EIS (xPH) H^+^, (h) EIS He^n+^, and (i) EIS O^n+^. Line spectra of differential flux for (j) EIS (TOF) H^+^ and (k) EIS He^n+^. (l) Vector magnetic field from DFG in GSM coordinates (note that since *Bx* < 0, −*Bx* is plotted for better visual comparison), (m) H^+^ flow speed from HPCA in GSM coordinates. Gaps in line flux data in Panels (j) and (k) are due to a lack of high‐energy‐resolution burst data; Panels (f)–(i) use lower‐energy‐resolution data during these periods. Panels (f)–(k) are combined spectra from MMS 2–4. Panels (l) and (m) are from MMS 3. Vertical black lines in (l) mark dipolarization‐like signatures.

Figure 2a shows a table of correlation coefficients of the time histories of flux for pairs of individual EIS energy channels of protons and helium (of an undetermined charge state) throughout the time period shown in Figures 1f–1m. The top row (in the uncolored region) lists the geometric mean energy of each H^+^ energy channel and the first column lists the geometric mean energy of each He^n+^ energy channel. The correlation coefficient table is color coded between 0 (red) and 1 (blue) to aid the eye toward regions of minimum or maximum correlation; negative correlation coefficients listed in the table are colored with the same dark red color as 0. Boxes with yellow outlines show helium and proton energy channels which are either closest to being equal (*E*_He_ = *E*_H_, as might be expected for He^+^) or where the helium energy is twice the proton energy (*E*_He_ = 2*E_H_, as might be expected for He^++^). One can readily see that there is a “ridge of high correlation” where the highest correlations (>0.7) are found for energies close to *E*_He_ = 2*E_H_. The correlations decrease rapidly on either side of this ridge. Note that there is very little correlation for *E*_He_ = *E*_H_. To further emphasize how good the correlations are between flux values where *E*_He_ = 2*E_H_, Figure 2c shows the intensity of H^+^ for three energy channels between 71 and 155 keV along with flux values of He^n+^ for energy channels between 149 and 310 keV. To best compare the flux responses, colors are kept the same for proton energy channels and helium energy channels with *E*_He_ = 2*E_H_. Furthermore, the He flux for each channel is both multiplied by a constant factor to bring its maximum flux in line with the maximum H^+^ flux during the time interval, and boxcar averaged over 1 min. In this comparison we see the remarkable coherency of the energy‐dispersed enhancements and reductions of energetic particle flux that are nearly identical for protons and helium with *E*_He_ = 2*E_H_. From these observations we conclude that the dominant charge state of helium is 2^+^, and that on average throughout the duration of this event the helium flux response is well ordered by *E/q*. A similar analysis of Figures 2b, 2d and 3e shows the the strongest component of oxygen ions have charges close to +6 for energies above 230 keV.

**Figure 4:**
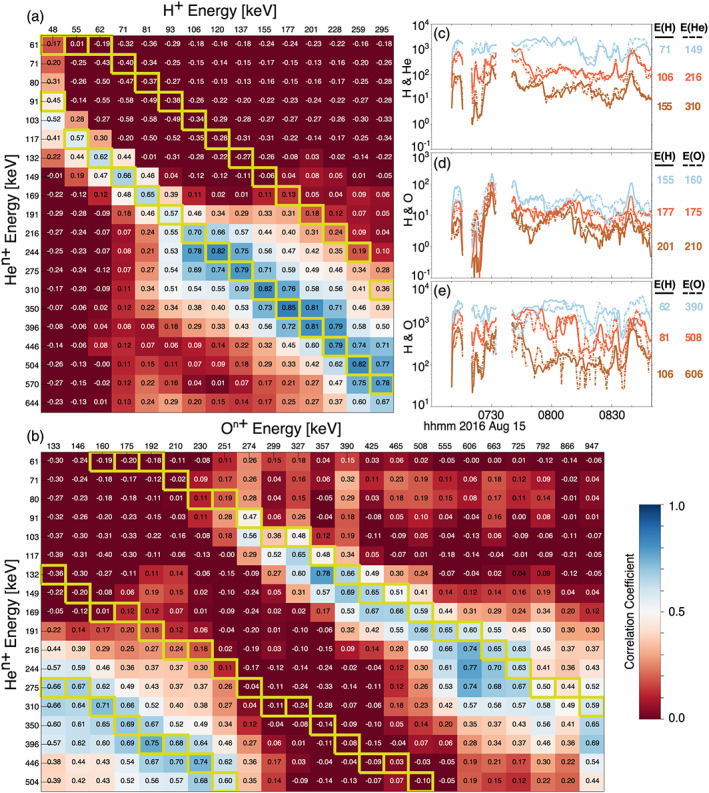
Panel (a) depicts correlation coefficients of time histories of EIS H and He flux between different energy channels of each species during the time period shown in Figure 3. Boxes with yellow outlines highlight energies where *E*_He_ = 2 * *E*_H_ and *E*_He_ = *E*_H_, which would indicate helium being He^++^ or He^+^, respectively. Similarly, Panel (b) depicts correlation coefficients between He and O, and yellow boxes highlight *E*_He_ = 2 * *E*_O_, *E*_He_ = *E*_O_, and 3 * *E*_He_ = *E*_O_, which with the inference from Panel (a) that helium is double ionized (He^++^) would signify oxygen charge states of 1, 2, or 6, respectively. Panel (c) shows the flux time histories of proton (solid lines) and helium (dashed lines) ions for energies *E*_He_ = 2 * *E*_H_. Each helium flux has been multiplied by a constant factor to visually compare its correlation with the proton fluxes. Similarly, Panel (d) shows proton (solid lines) and oxygen (dashed lines) fluxes for *E*_H_ = *E*_O_, and Panel (e) shows fluxes for 6 * *E*_H_ = *E*_O_.

Figure 3 shows the same parameters as Figure 1 for the period between 07:00 and 09:00 UT on 15 August 2016. As was the case for observations taken on 25 August 2016 shown in Figures 1 and 2, for this time period the MMS spacecraft are on the inbound leg of the orbit, sampling the premidnight region (at ~22 MLT) from 9.9 to 8.4 *R*_E_. Figure 4, like Figure 2, shows the correlation analysis between different energy channels of H^+^ with He (Figure 4a), He with O (Figure 4b), and normalized flux comparisons (Figures 4c–4e). Time gaps in the fluxes shown in Figures 3j–3k and 4c–4e stem from a lack of high‐energy‐resolution burst data during two short intervals; Figures 3f–3i show the lower‐energy‐resolution survey data during these intervals. Between ~07:00 and ~08:45 UT, the auroral indices (Figure 3e) indicate substorm activity as *AE* rises from ~60 to 460 nT, followed by a drop to ~350 nT. At MMS, energetic particle fluxes observed by EIS rapidly increased and HPCA observed intermittent fast flows from ~200–900 km/s in the earthward/duskward directions and ~100–400 km/s anti‐earthward. Like Figure 1, EIS observations are combined from MMS 2–4, and HPCA moments and magnetic field measurements are from MMS 3, but all spacecraft observe nearly identical features. Dipolarization‐like signatures in the magnetic field are observed at ~07:10 and ~07:22 UT.

The correlation table between H^+^ and He flux (Figure 4a) readily shows another well‐organized ridge of high correlation between H^+^ fluxes and He^n+^ fluxes for energies where *E*_He_ = 2*E_H_, and a weak correlation for most other energies. Again, this result is indicative that the helium being observed is He^++^. Comparing helium and oxygen (Figure 4b), there are now two strong ridges of high correlation with regions of low correlations on either side of them. The ridges of high correlation are found for oxygen energies between ~130–250 keV where *E*_He_ = 2*E_O_ and for oxygen energies >250 keV where 3*E_He_ = *E*_O_. Using the deduced helium charge state of 2, if the flux response is ordered by *E/q*, this implies that the lower‐energy oxygen range is mostly singly charged and the higher‐energy range is 6^+^. While not shown, the correlation table between H^+^ and O^n+^ shows the same trend and leads to the same deductions of charge state. Like the previous case in Figure 2, the comparisons of normalized oxygen and helium fluxes with H^+^ for the same *E*/*q* show a good coherency between flux enhancements and reductions (Figures 4c–4e). Furthermore, comparing energies *E*_O_ = *E*_H_ for this case (Figure 4d) shows a much better agreement and consistent coherency than in the previous case (Figure 2d). This further indicates that the 25 August 2016 case (Figures 1 and 2) has a mixture of charge states (i.e., no clearly dominant one) below ~230 keV for oxygen, while this second case from 15 August 2016 shows clearly separable dominant charge states of oxygen, with O^+^ dominating below ~250 keV and O^6+^ dominating at higher energies.

**Figure 5:**
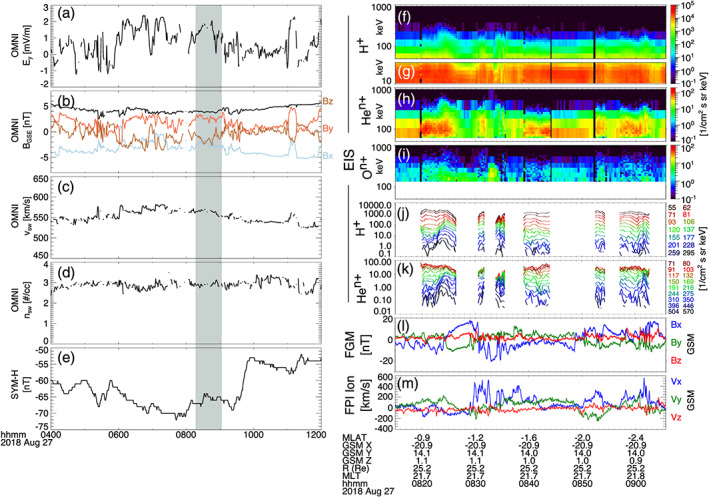
Solar wind observations from the OMNI database in GSE coordinates (a–d), auroral electrojet *AE*, *AU*, and *AL* indices (e), and MMS observations (f–m) of energetic ion enhancements during substorm activity. Solar wind (a) *y* component of electric field, (b) vector magnetic field, (c) speed, and (d) density. Energy spectra of differential flux for (f) EIS (TOF) H^+^, (g) EIS (xPH) H^+^, (h) EIS He^n+^, and (i) EIS O^n+^. Line spectra of differential flux for (j) EIS (TOF) H^+^ and (k) EIS He^n+^. (l) Vector magnetic field from DFG in GSM coordinates, and (m) ion flow speed from FPI in GSM coordinates. Gaps in line flux data in Panels (j) and (k) are due to a lack of high‐energy‐resolution burst data; Panels (f)–(i) use lower‐energy‐resolution data during these periods. Panels (f)–(k) are combined spectra from MMS 2–4. Panels (l) and (m) are from MMS 3.

By 2018, the apogee of the MMS constellation was raised to ~25 *R*_*E*_, which allows us to also look at cases of energetic particle enhancements deeper in the tail. Due to sharper changes in the magnetic field deeper in the tail, one might not expect strong coherency of particle fluxes between ions with different gyroscales. Following a similar format as Figures 1 and 3, Figure 5 shows an event on 27 August 2018 when MMS was located at ~22.7 MLT and ~25.2 *R*_*E*_. Solar wind observations from OMNI (Figures 5a–5d) and the geomagnetic SYM‐H index (Figure 5e) are provided from 04:00–12:00 UT for context. SYM‐H is shown in place of *AE* (as in Figures 1 and 3) as only “real‐time” *AE* data were available at the time of publication. The period shown is during the recovery phase of geomagnetic storm which had a minimum *Dst* of −174 nT at 08:00 UT on 26 August 2018. From 08:00–09:15 UT on 27 August 2018, EIS energetic ion data from MMS 2–4 (Figures 5f–5k), FGM magnetometer data from MMS 3 (Figure 5l), FPI‐DIS total ion flow speeds from MMS 3 (Figure 5m) are shown.

**Figure 6:**
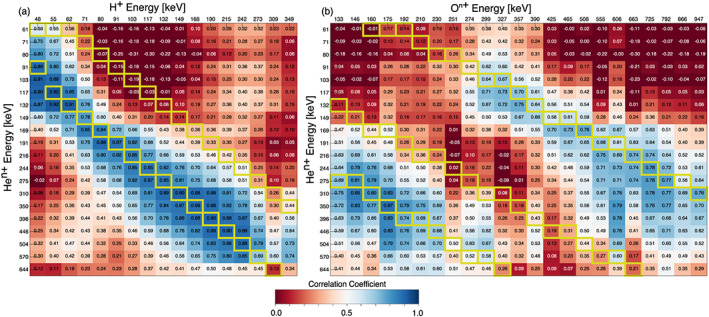
Panel (a) depicts correlation coefficients of time histories of EIS H and He flux between different energy channels of each species during the time period shown in Figure 5. Boxes with yellow outlines highlight energies where *E*_He_ = 2*E_H_ and *E*_He_ = *E*_H_, which would indicate helium being He^++^ or He^+^, respectively. Similarly, Panel (b) depicts correlation coefficients between He and O, and yellow boxes highlight *E*_He_ = 2*E_O_, *E*_He_ = *E*_O_, and 3*E_He_ = *E*_O_, which with the inference from Panel (a) that helium is double ionized (He^++^) would signify oxygen charge states of 1, 2, or 6, respectively.

Flux correlation tables between H^+^ and He^n+^, and He^n+^ and O^n+^ (Figures 6a and 6b, respectively) are presented in Figure 6. It should be noted that during this event the raw derived *AE* index was between ~500–750 nT, indicating substorm activity. FPI observed multiple periods of primarily earthward directed BBFs ranging between ~200–600 km/s from ~08:29–09:05 UT and duskward/partially tailward flow between 50 and 200 km/s from ~08:15–08:27 UT. The magnetic field signatures show multiple crossings of the plasma sheet, indicated by reversals in *B*_*x*_. When the MMS constellation is closer to the central plasma sheet (lower |*B*_*x*_|) and concurrent with fast flows, large enhancements of energetic ions are observed for energies up to ~300 keV for protons, ~600 keV for helium, and ~1 MeV for oxygen. As shown in Figure 6, even as MMS traverses in and out of a highly dynamic region in the tail, there is still a high degree of correlation (>0.7) between the protons and helium for energies *E*_He_ = 2*E_H_ (suggesting He^++^). Similarly, there is high correlation between oxygen and helium energies *E*_He_ = 2*E_O_ for oxygen energies below ~250 keV (suggesting O^+^ and He^++^), and 3*E_He_ = *E*_O_ for oxygen energies above ~300 keV (suggesting O^6+^ and He^++^). Again, these distinct ridges of high correlation seem indicative that the helium present is He^++^, oxygen below ~250 keV is O^+^, oxygen above ~300 is O^6+^. The 250 to 300 keV range represents a transition in oxygen charge state dominance from O^+^ to O^6+^.

## 4 Discussion

We have shown with case studies using MMS observations that during periods of enhanced energetic ion injections the highest energies of ions (≳250 keV) are not protons, but rather are helium and oxygen. This condition holds in cases from both the near‐Earth and distant regions of the magnetotail shown. Additionally, it holds for over 40 individual cases that we have examined, of which, the three cases shown here are representative. This conclusion is contrary to the commonly held assumption that protons are the dominant species observed by a species‐indiscriminate charged particle instrument at all energies (e.g., Blake et al., 2016; Mauk, 2013). The present paper is not the first to make such a claim. Rather, it continues a history of high energy observations of heavy ions at higher intensities than protons, and adds substantial new details about how the energization is organized. We show that a correlation technique introduced by Mitchell et al. (2018) is able to illuminate the most likely dominant heavy ion charge states by comparing their fluxes with that of a fiducial species. This analysis shows that the highest energy ions are most likely multiply charged ions of solar wind origin. While these high energy solar wind heavy ions are potentially only minor players in the global electrodynamic system, understanding their presence and energization history has large potential impacts on understanding the energization and transport mechanisms in the magnetosphere.

One consequential impact is that, absent knowledge of the presence of these different ion mass and charge species, researchers are more inclined to introduce a mass‐discriminating acceleration mechanism. Such mechanisms sometimes invoke differences in these ion species' respective gyromotions in the tail, allowing O^+^ to be energized hundreds of keV more than H^+^ (e.g., Delcourt et al., 1997; Greco et al., 2015). For regions just earthward of the magnetotail, Mitchell et al. (2018) posited their findings of charge‐state‐dependent energization of ion injections as potential evidence of purely adiabatic ion energization in injections with a simple flow channel injection model. However, other theoretical studies have shown that a charge‐state‐dependent energization and high temporal correlation do not necessarily imply adiabaticity (Catapano et al., 2017; Ukhorskiy et al., 2018). The results from the study presented here cannot determine the dominant mechanism of ion energization in the tail, other than to show that it roughly orders the energization according to *E*/*q*. These results show that during injections, an electric field affects the suprathermal plasma in a manner that is primarily insensitive to gyroradius and gyrofrequency, contrary to mechanisms that posit a mass‐dependent energy gain due to differences in gyromotion (e.g., Delcourt & Sauvaud, 1994; Greco et al., 2015; Kronberg et al., 2015; Nosé et al., 2001; Zelenyĭ et al., 2007). We cannot say if mass‐dependent energization processes might take place elsewhere. We claim only that the suprathermal to energetic plasma observed during these events, examined throughout different regions of the magnetotail, have little evidence of a mass‐dependent energization. For these events, mass‐dependent mechanisms are most likely not the dominant mechanism for the highest energy heavy ions found in the magnetosphere (i.e., ~1 MeV oxygen).

**Figure 7:**
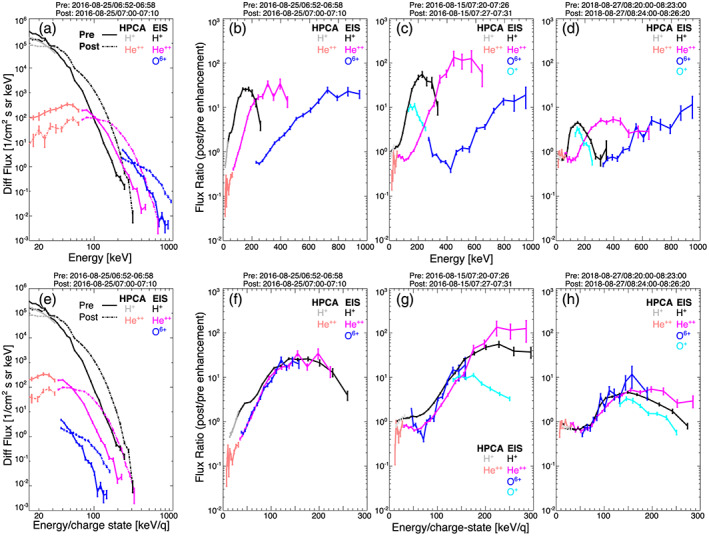
Panels (a) and (e) depict average fluxes versus energy and energy per charge, respectively, for preenhancement (solid lines) and postenhancement (dashed lines) time periods (denoted above each panel). Panels (b)–(d) and (f)–(h) depict flux ratios (preenhancement/postenhancement) versus energy and energy per charge, respectively, for the time periods listed above each panel. Panels (a), (b), (e), and (f) are from an enhancement during the event shown in Figure 1. Panels (c) and (g) are from an enhancement shown in Figure 3. Panels (d) and (h) are from an enhancement shown in Figure 5. In each panel, black (EIS) and gray (HPCA) are H^+^, magenta (EIS) and peach (HPCA) are He^++^, dark blue is O^6+^ (EIS), and light blue is O^+^ (EIS). The charge state of a given EIS heavy ion is deduced from Figures 2, 4, and 6.

Using MMS/EIS, Cohen et al. (2017) showed that the average He^n+^ and O^n+^ fluxes dominated the H^+^ fluxes above ~150–220 keV. They hypothesized that the helium and oxygen charges states were *n* = 2 and *n* = 6, respectively, in part because their average He^n+^ and O^n+^ spectra showed a good visual agreement with H^+^ when plotted as intensity versus *E/q* for these charge states. Additionally, these charge states and spectral shapes were consistent with what was reported by AMPTE/CCE/CHEMS and AMPTE/IRM/SULEICA, and Polar/CAMMICE‐MICS (Allen et al., 2017; Allen, Livi, & Goldstein, 2016; Allen, Livi, Vines, & Goldstein, 2016; Gloeckler & Hamilton, 1987; Kremser et al., 1987). Mobius et al. (1987) reported that the presubstorm and postsubstorm spectra for H^+^, He^+^, He^++^, O^+^, and CNO^≥2+^ were both best ordered by *E/q*. They found that associated with the hardening of the spectrum from the substorm, the ratio of flux enhancement was also well ordered by *E/q*. However, ionospheric O^+^ received a slightly greater level of enhancement than other species. Figure 7 shows an example of the change in the spectrum during a flux enhancement and examples of the ratio of flux enhancement during each of the events shown here. Figures 7a and 7e compare the average preenhancement and postenhancement differential flux versus *E* and *E*/*q*, respectively, of an energetic particle enhancement on 25 August 2016 between 06:25 and 06:58 UT (pre) and 07:00:07:10 UT (post), which take place during the event shown in Figures 1 and 2. In order to extend to lower energies, HPCA H^+^ and He^++^ flux are also shown. Here we use the deduced *q* for a given EIS energy channel from Figure 2 (since it is unclear what the charge state of <230 keV oxygen is, only energies above 230 keV are shown in Figures 7a and 7e). Error bars at a given point represent the one standard deviation error $\left(\frac{1}{\sqrt{n}}\right)$ of the flux. One can readily see that in Figures 7a, when flux is plotted versus energy, heavy ion fluxes dominate proton fluxes above ~120–200 keV. Additionally, while proton flux is enhanced above ~30 keV, helium flux is only enhanced above ~100 keV, and oxygen is only enhanced above ~280 keV. However, when plotted versus *E*/*q* (Figure 7e), the spectral shape of both the preenhancement and postenhancement flux is well aligned, and the ranges of *E*/*q* which have a flux enhancement show a better agreement for all species. An *E/q* ordering of the enhancement ratio is captured in the earlier figures discussed, specifically Figures 2c–2e and 4c–4e. In those plots, the heavy ion fluxes are multiplied by one constant factor for each energy channel to bring it up to the peak H^+^ flux, and the peaks and valleys follow each other well for both species. Hence, the ratios of flux enhancement are expected to be the same for these species. To better illustrate this finding, Figures 7b–7d and 7f–7h compare the ratio of flux enhancement versus *E* and *E*/*q*, respectively, during a time period in each event previously shown in Figures 1, 2, 3, 4, 5, 6. Here the charge state used for a given energy channel is determined by our correlation analyses shown in Figures 2, 4, and 6. We use O^+^ for *E* ≤ 251 keV and O^6+^ for *E* ≥ 274 keV for Figures 7c and 7g, and O^+^ for *E* ≤ 230 keV and O^6+^ for *E* ≥ 327 keV for Figures 7d and 7h. It is readily apparent that the ordering of the flux ratios is significantly better by *E/q* than by *E*. This *E*/*q*‐dependent ordering is suggestive of an electric field acceleration mechanism which is mass independent. Additionally, it should be noted that in Figures 7b and 7c O^+^ does not have a greater ratio of flux enhancement compared to the other ions, contrary to a finding of Mobius et al. (1987). But, as reported in Ono et al. (2009), individual enhancement events can often have a greater spectral hardening in either H^+^ or O^+^.

It is worth noting how remarkably well correlated the flux responses are between different ions species during such seemingly transient periods (large flows, quickly changing fields, rapid enhancements, etc.). A potentially important point from the coherency of the flux enhancement and reduction is that often MHD and test particle simulations of dipolarizations and injections show rather complex particle motion within and near these fronts (e.g., Birn et al., 2015). The gyroradii of energetic O^+^, for example, are so large that the behavior of these ions is highly uncertain and perhaps chaotic. Two different ions measured at a given time at a local position can come from vastly different regions of the magnetotail. If so, one would expect that the energy received by such ions would depend strongly on their source positions. Such a condition would make it unlikely to find a high degree of correlation and consistency between the intensity of different species with different charge states. Thus, our finding, particularly for the more distant regions of the magnetotail, could have implications for the spatial and temporal scales of energization and the local field structure. We will also note that Ipavich and Scholer (1983) showed with observations from ISEE 1 that there was an energy dependence of the decay of the highest energies comprising the suprathermal ion component of the plasma sheet, where higher energies dropped in flux earlier in time than lower energies. Our results in Figures 2c–2f and 4c–4f show that the dispersion in the reductions of flux are well ordered by *E/q*.

Since TOF‐SSD instruments, like MMS/EIS and the Van Allen Probes/RBSPICE, have flown on many missions in the past, this correlation technique can most likely be applied to many other datasets during these types of flux enhancements across a wide range of plasma conditions or even other planetary magnetospheres. For instance, a similar approach has recently been applied to Juno/JEDI dataset to identify the dominant charge states of high energy heavy ions (oxygen and sulfur) in Jupiter's magnetosphere (Clark et al., 2020).

## 5 Conclusions

In this study, we have used MMS/EIS observations at different radial distances within the nightside magnetotail of substorm‐related energetic ion enhancements concurrent with and near BBFs to show that

1. At high suprathermal energies (*E* ≳ 200–300 keV) primarily heavy ions (helium and oxygen) are observed with very little proton flux.
2. The charge state of heavy ions in these dynamic regions can be inferred using a temporal correlation analysis with a fiducial species when only mass composition is directly measured.
3. The higher‐energy suprathermal heavy ions are primarily multiply charged (He^++^ and O^6+^).
4. The energization and dispersion of heavy ions in energetic particle injections is ordered by charge state such that using the deduced charge state for a given species (H^+^, He^++^, O^+^, and O^6+^) and energy give a flux‐versus‐time response that is remarkably coherent for constant *E*/*q* over extended periods of time within highly dynamic regions of the magnetotail.
5. The ratio of preenhancement to postenhancement fluxes is well ordered by *E/q*.

Since the suprathermal ion population can comprise a significant contribution to the energy density of the plasma sheet and ring current, this study highlights the importance of understanding ion mass composition and charge state while evaluating the source, transport, and energization of suprathermal ions. While we show flux responses that are remarkably well ordered by *E*/*q*, multiple different causal mechanisms have been put forward which could generate such a response. For example, as noted earlier, ion drift across an ideal flow channel with an enhanced electric field will adiabatically produce charge‐dependent energization and *E*/*q‐*dependent drift trajectories (Mitchell et al., 2018). Test particle simulations of ion interactions with stochastic electromagnetic fluctuations, like those accompanying dipolarization fronts in the magnetotail, have shown primarily charge‐dependent energization with only a small mass dependency (Catapano et al., 2017). Additionally, test particle MHD simulations have suggested that magnetic islands can form within dipolarization fronts and lead to circumferential gradient drifts around the island and nonadiabatic charge‐state‐dependent energization (Ukhorskiy et al., 2018). Future work is still needed to identify their relative roles in ion energization. Additionally, while we show evidence that the highest energies of oxygen observed (≳300 keV) are of solar wind origin which obtains a higher energy due to its higher charge state, future work and observations are still needed to confine and identify the potential role of nonadiabatic acceleration of O^+^ and differences in O^+^ energization compared to other species.

## Data Availability

The MMS data used in this work are publicly available Level 2 data and can be retrieved from the MMS Science Data Center (at https://lasp.colorado.edu/mms/sdc/). The solar wind data and geomagnetic index data can be retrieved online (from https://omniweb.gsfc.nasa.gov/).
